# A conserved transcriptional fingerprint of multi-neurotransmitter neurons necessary for social behavior

**DOI:** 10.1186/s12864-022-08879-w

**Published:** 2022-09-29

**Authors:** Denver Ncube, Alexandra Tallafuss, Jen Serafin, Joseph Bruckner, Dylan R. Farnsworth, Adam C. Miller, Judith S. Eisen, Philip Washbourne

**Affiliations:** Institute of Neuroscience, 1254 University of Oregon, Eugene, OR 97403 USA

**Keywords:** Neurotransmitter, Social behavior, Neuronal identity, Transcription factors

## Abstract

**Background:**

An essential determinant of a neuron’s functionality is its neurotransmitter phenotype. We previously identified a defined subpopulation of cholinergic neurons required for social orienting behavior in zebrafish.

**Results:**

We transcriptionally profiled these neurons and discovered that they are capable of synthesizing both acetylcholine and GABA. We also established a constellation of transcription factors and neurotransmitter markers that can be used as a “transcriptomic fingerprint” to recognize a homologous neuronal population in another vertebrate.

**Conclusion:**

Our results suggest that this transcriptomic fingerprint and the cholinergic-GABAergic neuronal subtype that it defines are evolutionarily conserved.

**Supplementary Information:**

The online version contains supplementary material available at 10.1186/s12864-022-08879-w.

## Background

Common convention classifies neuronal subtypes and predicts their functions based on a single neurotransmitter (NT). The limitation of this approach is that it assigns neuronal identity based on the first NT to be detected in a neuron, and often the possibility of other NTs in neurons of interest are not investigated. However, with the prevalence of single cell sequencing atlases and wide-scale studies, there is increasing evidence that many neurons have the capacity to synthesize and release more than one NT [1–3]. To understand how neural circuits function it is critical to know the exact nature of the signal being sent from one neuron to the next. Which NT is interacting with what type of receptor at a given synapse? Furthermore, the multi-transmitter phenotype has important implications in relation to pharmacological agents that alter the release of NTs. Some classes of drugs are designed based on a single NT released by a specific neuronal type, ignoring other potential NTs they may also release. Potential outcomes of such a limited approach include a reduced efficacy of pharmacological agents and an increase in unintended consequences [4].

Neurons can release multiple transmitters via different mechanisms. Co-transmission can be broadly defined as the release of multiple NTs from non-overlapping pools of synaptic vesicles [5] and co-release happens when the NTs are released from the same pool of synaptic vesicles [6]. Co-transmission is quite common, in which release of a “classical” small molecule NT [glutamate, GABA, glycine, acetylcholine (ACh)], is accompanied at the same synapse by release of neuropeptides such as somatostatin, neuropeptide Y, substance P and enkephalin (among many others) [7]. The neuropeptides have much slower effects on the properties of target neurons. In addition, classical NTs can be accompanied by monoamine (serotonin, dopamine, adrenaline and noradrenaline) and purine NTs (adenosine and ATP) at the same synapse, often modulating the effect of the classical NTs ([8]: [9]).

In addition to releasing both classical and neuropeptide NTs, many neuronal subtypes have been shown to release multiple classical NTs [10]. In some cases, the released NTs have similar post-synaptic effects, for example inhibition mediated by GABA and glycine from spinal interneurons [11]. In other instances, the co-released NTs might have antagonistic effects, such as GABA and ACh release by mouse striatal neurons [12]. To accurately characterize multi-transmitter neurons, emphasis must be placed on using genetic and protein markers that are relevant to the synthesis and packaging of specific NTs [13]. Utilizing such an approach provides an accurate identification and characterization of neurons by establishing the presence of the critical machinery required to synthesize, package, or release a specific NT.

In this study, we adopted this approach, and utilized molecular techniques to transcriptionally profile vTel^y321^ neurons, a cluster of forebrain neurons that modulate zebrafish social behavior. A shelf screen of telencephalic driver lines identified the enhancer trap Et^y321^, whose genetically-defined neuronal population is necessary for social orienting and approach in a split dyad assay [14]. We previously showed that vTel^y321^ neurons are cholinergic [14], but here we combine transcriptomic, in situ hybridization (ISH) and immunohistochemistry (IHC) experiments to reveal that these neurons are also GABAergic. The multi-NT identity is detectable during early embryonic development and identity acquisition is not sequential; rather, machinery for both transmitters is expressed simultaneously. We also discover a combination of LIM Homeobox transcription factor genes that are reliable markers of these neurons in embryos, larvae, and adults. Our analysis provides a “transcriptomic fingerprint” for this cluster of forebrain neurons that we utilize to identify homologous clusters of neurons in rodents. This genetic congruity supports the conclusion that vTel^y321^ neurons are evolutionarily conserved and will be useful to determine whether the transcriptional similarity underlies functional homology.

## Results

### vTel^y321^ transcriptome confirms its transgenic origin

**Fig. 1:**
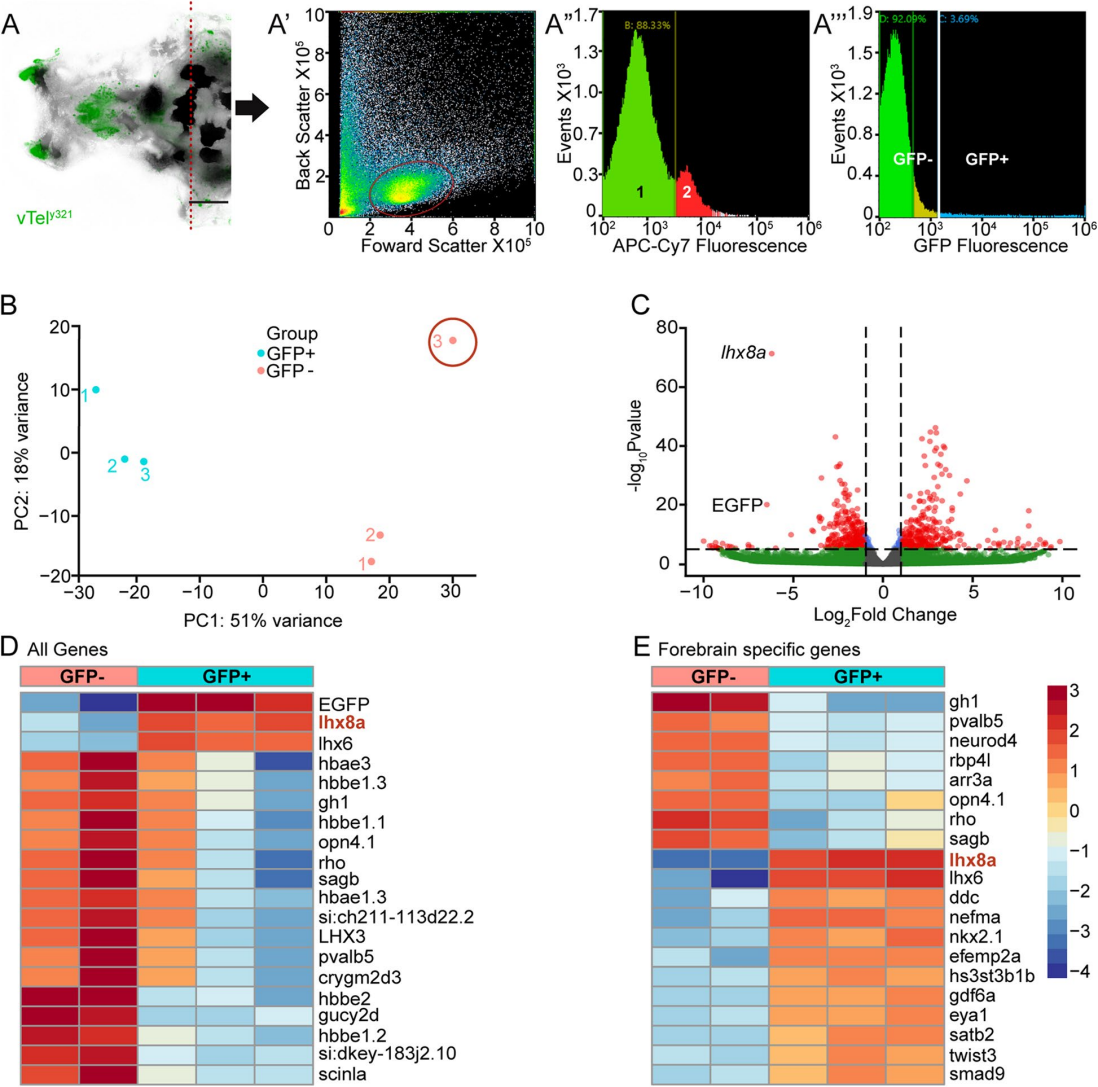
vTel^y321^ transcriptome confirms its transgenic origin. **A** Dorsal view of *vTel*^*y321*^ transgene expression in the forebrain and the dissection plane (dotted red line) for dissociation. Overlay of fluorescence (green) and transmitted light (greyscale). Scale bar = 100 μm. A’ FACS isolation of cell cluster (circled) after dissociation. A” Graph shows cutoff between live (1, green) (88.33%), and dead (2, red) cells (11.67%). A”’ Proportions of GFP positive (cyan, 3.69%) and negative cells (green, 92.09%) from the live cell fraction. **B** Principal component analysis of GFP positive and negative samples after variance stabilization transformation of gene count data. Sample 3 in the GFP negative fraction was dropped from subsequent analysis as an outlier. **C** Volcano plot of the ~ 23,000 genes expressed in the samples. Gray: Not significantly enriched and/or no fold change; Blue: significant differential expression (*p* < 0.05) but with fold change below threshold; Green: Up or downregulated (positive or negative fold change) without significant differential expression; Red: Significantly differentially regulated**. D** Heatmap of the 20 genes with the greatest differential expression between GFP positive and GFP negative samples. **E** Heatmap of the 20 forebrain specific genes with the greatest differential expression between GFP positive and GFP negative samples

We previously showed that manual and chemogenetic ablations of vTel^y321^ neurons disrupts zebrafish social orienting behavior [14], prompting us to further characterize this cell population. vTel^y321^ neurons are genetically defined by the enhancer trap insertion *Et (rex2-scp1:gal4ff)y321* (Fig. 1a) and predominantly lie within the ventral nucleus of ventral telencephalon (Vv) and dorsal nucleus of ventral telencephalon (Vd) of the zebrafish telencephalon. To learn the molecular identity of this population of neurons, we dissected heads of 7-day post-fertilization (dpf) larvae (*n* = 80) to isolate as much of the telencephalon as possible (Fig. 1A). After dissociation, we used fluorescence-activated cell sorting (FACS) to sort and pool GFP positive and negative cells (Fig. 1A’). 80–90% of dissociated cells were alive (Fig. 1A”) and 3.69% of the cells were GFP positive (Fig. 1A”’). We expected the proportion of GFP positive cells to be small because the vTel^y321^ nucleus is a relatively limited portion of the telencephalon, totaling several hundred neurons at this stage [15]. Sorting was performed until at least 100,000 cells were collected for mRNA purification and sequencing of both the GFP positive and negative samples. We used Ensembl to select protein-encoding genes and compiled lists of genes, ordered by adjusted *P* values, that are differentially expressed (DE) between GFP positive and GFP negative cells. Principal component analysis revealed that one sample from the GFP negative fraction was an outlier (Fig. 1B). This sample was dropped leaving us with 3 GFP positive samples and 2 GFP negative samples. Sorting for genes with an adjusted *P* value ≤0.05 generated a list of 2096 DE genes expressed in GFP positive cells compared to GFP negative cells out of a total of ~ 23,000 genes (9%, Fig. 1C).

To refine our characterization of vTel^y321^ neurons, we focused on genes documented to be expressed in the forebrain by the Zebrafish Information Network (ZFIN; zfin.org), compiling a list of 454 genes (21.6% of the 2096 DE genes). When we reviewed the top 20 differentially expressed genes from the entire dataset and the top 20 forebrain genes (Fig. 1D, E), we discovered that the LIM transcription factor (LIMTF) encoding gene *lhx8a* (Fig. 1C) was highly enriched in the GFP positive population of neurons. We had expected to observe significant enrichment of *lhx8a* in GFP positive neurons since the enhancer trap *Et*^*y321*^ is located within the *lhx8a* gene locus (Zebrafish Brain Browser, zbbrowser.org) (Additional Table 1). Taken together with the high enrichment of EGFP transcripts in the GFP positive population (Fig. 1D), we conclude that the dataset successfully represents an enriched population of mRNAs from the FACS sorted cells.

### vTel^y321^ neurons express two classical NTs

**Fig. 2:**
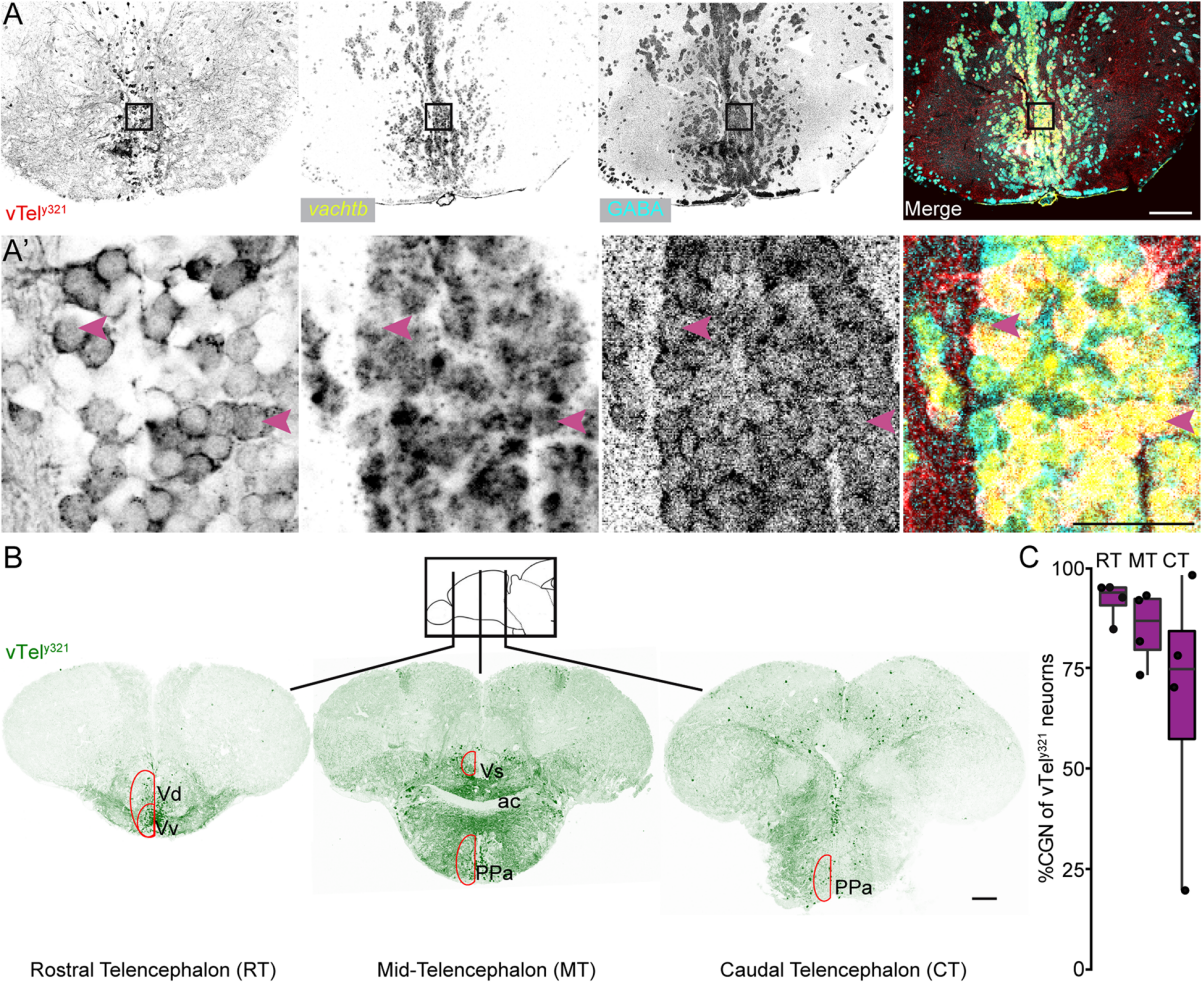
vTel^y321^ neurons are both cholinergic and GABAergic. **A** Coronal sections of the adult ventral forebrain in the rostral telencephalon showing expression of the* Et* *y321* transgene-dependent GFP, mRNA expression of *vachtb* and GABA immunolabeling. **A’** Insets of boxed areas in **A**. Arrowheads point to selected vTel^y321^ neurons that express *vachtb* and GABA (Scale bars: A = 100 μm; A’ = 50 μm). **B** Coronal sections of ventral forebrain depicting some nuclei in the forebrain that include vTel^y321^ neurons and which provide anatomical positions used for rostro-caudal plane identification prior to quantification of vTel^y321^ CGNs (RT, rostral telencephalon; MT, mid-telencephalon; and CT, caudal telencephalon; Vv, Ventral nucleus of the ventral telencephalon; Vd, dorsal nucleus of the ventral telencephalon; Vs, supracommisural nucleus; PPa, Parvocellular pre-optic nucleus; ac, anterior commissure) (scale bar: B = 100 μm). **C** Boxplots showing percentage of adult vTel^y321^ neurons that are cholinergic and GABAergic at selected anatomical forebrain landmarks. *N* = 4

The rodent *lhx8a* homolog, *Lhx8*, is required for development and function of cholinergic neurons in the rat forebrain [16, 17], and we previously showed that vTel^y321^ neurons are cholinergic [14]. Consistent with our previous observations, ISH confirmed detection of transcripts for cholinergic markers such as the vesicular acetylcholine transporter (*vachtb, n* = 4) (Fig. 2A, A’) and choline acetyltransferase (*chatb, n* = 4) in vTel^y321^ neurons (Additional Fig. 1). In addition, we also found significant differential expression of *slc5a7,* encoding the choline transporter, further validating the cholinergic identity of these neurons. We compiled a list of NT markers that were expressed, and established which ones were significantly DE (Table 1). Given previous descriptions of large numbers of GABAergic neurons in the subpallium [18, 19], it was not surprising to discover that, in addition to cholinergic transcripts, vTel^y321^ neurons also express several GABAergic specific transcripts including *slc32a1* (the vesicular GABA transporter, *vgat*) and glutamate decarboxylase encoding genes *gad2* and *gad1b* (Table 1), suggesting that vTel^y321^ neurons are both GABAergic and cholinergic.

**Table 1:**
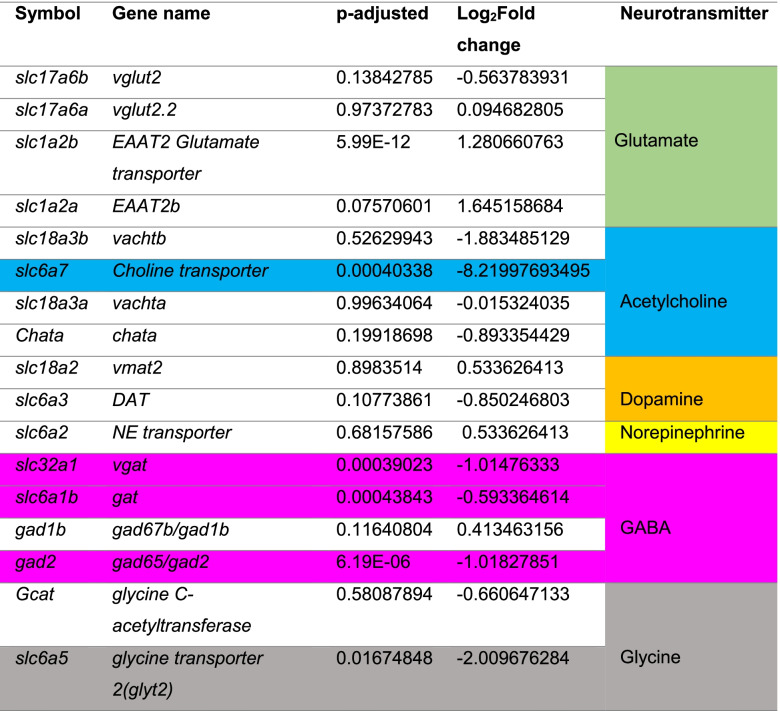
Neurotransmitter-associated genes expressed in vTel^y321^ neurons

Highlighted genes are significantly DE with enhanced expression in vTel^y321^ neurons denoted by an adjusted *p*-value (*p* < 0.05) and negative Log_2_Fold change. The letters *slc* denote genes belonging to the solute carrier superfamily of genes

To determine if individual vTel^y321^ neurons are cholinergic-GABAergic neurons (CGNs), we combined GABA immunolabeling and ISH for *vachtb* or *chatb*. We found that vTel^y321^ neurons are both cholinergic and GABAergic (Fig. 2B) based on expression of both *vachtb* (Fig. 2A) and *chatb* (Additional Fig. 1) overlapping with GABA labeling in sections of adult telencephalon. Approximately 81% of GFP positive cells were both GABAergic and cholinergic across all anatomical locations examined (Fig. 2B, C and Table 2). However, we noticed that the proportion of vTel^y321^ neurons that were CGNs was much higher in the rostral telencephalon (RT, ~ 92%), and the proportion progressively decreased toward the caudal region of the ventral telencephalon (Fig. 2C, Table 2). In contrast, only 8.7 and 14.9% of vTel^y321^ neurons were positive for exclusively GABA or *vachtb*, respectively, across all locations examined (Table 2). This proportion was much lower in the RT, and progressively increased in a caudal direction. We conclude that although the proportion of vTel^y321^ neurons that are CGNs is consistently large across the telencephalon, this proportion decreases in the rostral to caudal direction with the proportion of single neurotransmitter neurons increasing along the same axis.

**Table 2:**
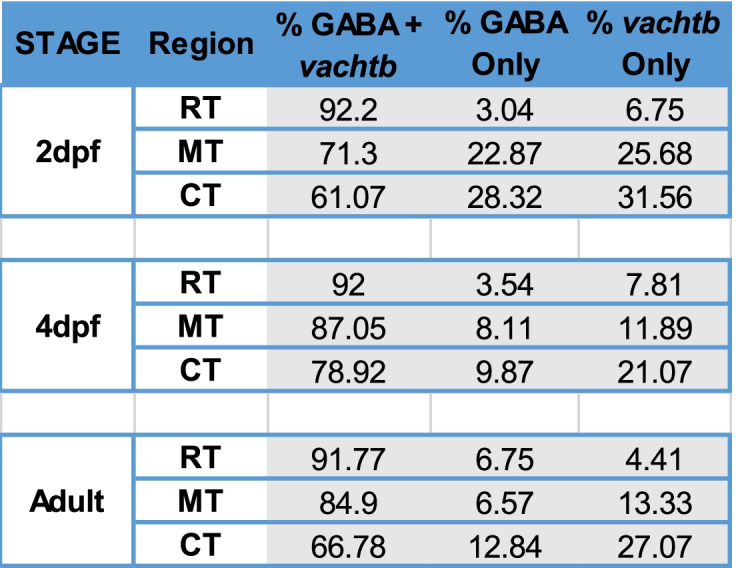
Proportions of vTel^y321^ neurons expressing NT phenotypes

### The CGN phenotype is constant throughout development

**Fig. 3:**
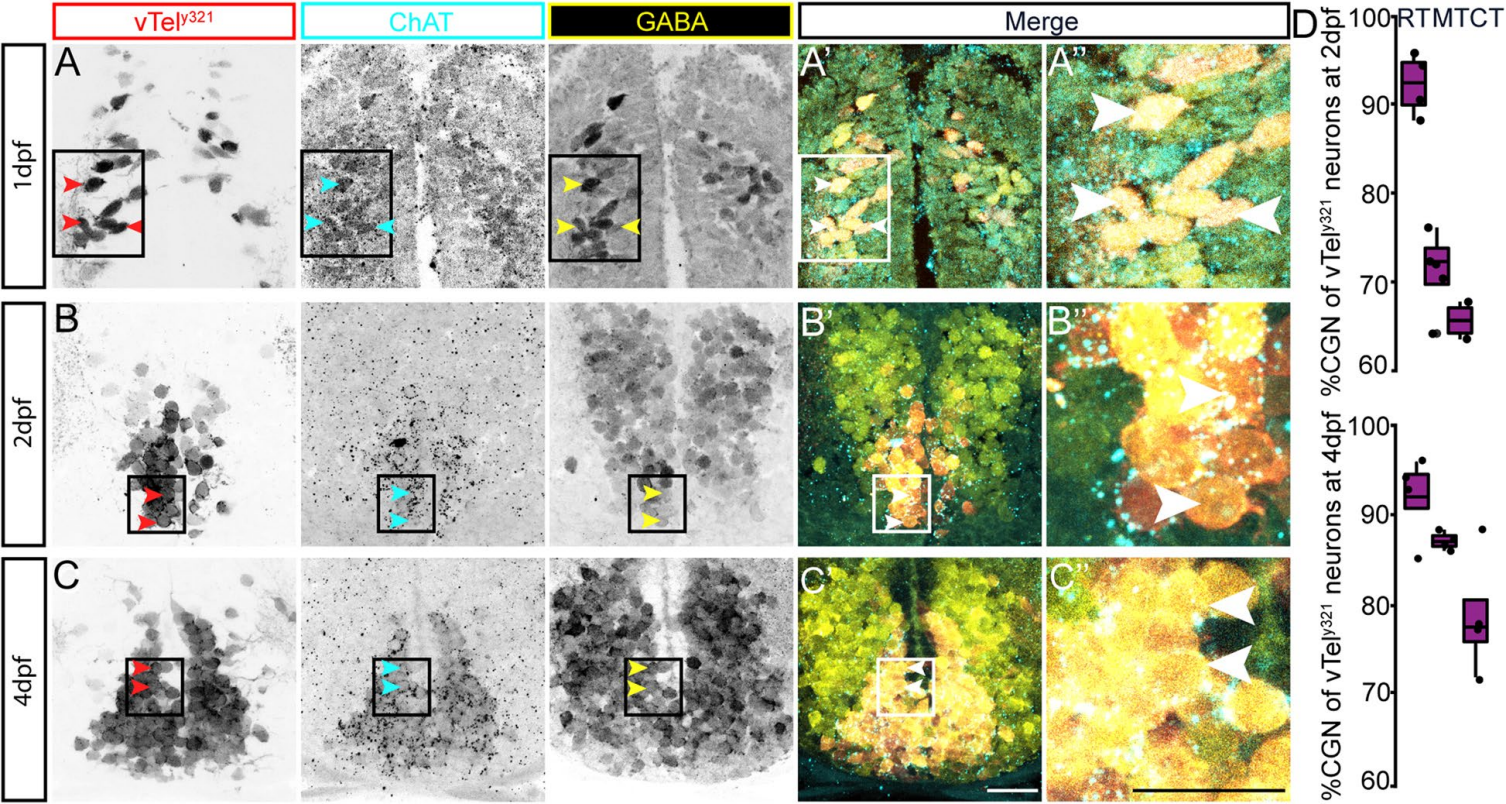
CGN phenotype is constant throughout development. Expression of cholinergic and GABAergic markers between 1 and 4 dpf. **A** Transverse section at 1 dpf showing expression of cholinergic (ChAT) and GABAergic (GABA) immunolabeling. A’, A”, B′, B″, and C′, C″. Rectangular boxes in A’ define selected areas, shown at higher magnification in A”, containing neurons labeled with the respective image color. Red arrows label vTel^y321^ neurons, cyan arrows cholinergic vTel^y321^ neurons, and yellow arrows label GABAergic vTel^y321^ neurons. Merged images showing vTel^y321^ neurons labeled (white arrows) that are cholinergic and GABAergic from 1dpf to 4dpf. A”, B″ and C″ inserts from areas defined in A’, B′ and C′. Arrows in merged images A’, B′, and C′ and inserts A”, B” and C” show selected vTel^y321^ neurons that are cholinergic and GABAergic. **B**, **C** Coronal sections of ventral telencephalon showing cholinergic and GABAergic markers in vTel^y321^ neurons at 2 and 4 dpf. (Scale bar for all images *=* 20 μm). **D** Boxplots showing the percentage of vTel^y321^ neurons that are CGNs at 2 and 4 dpf

Previous studies suggest that NT fate acquisition could be sequential, in which neurons first develop as single NT neurons and later express an additional NT [20–23]. To learn whether vTel^y321^ neurons develop their cholinergic and GABAergic fates in a particular order, or whether that order differed along the telencephalic axis of these neurons, we examined when they attained their multi-NT phenotype, and whether it occurred sequentially. If the CGN phenotype was attained in a sequential manner, we would expect to see varying proportions of vTel^y321^ neurons that were CGNs at different time points. To determine if this was the case, we crossed *Et*^*y321*^ [*Et (rex2-scp1:gal4ff)y321*] to *Tg (UAS:GFP)* fish, raised the resultant F1 embryos, and performed IHC labeling for cholinergic and GABAergic markers at multiple developmental stages. These experiments enabled us to determine that the *y321* transgene begins driving GFP expression in vTel^y321^ neurons at 24 hpf. This was also the earliest timepoint we could detect GABAergic and cholinergic markers (Fig. 3A, A’, 3A”), and the labeling was consistent across development (Fig. 3A, A’, 3A”, 3B, 3B’, 3B″, 3C, 3C’, 3C″). These results suggest that CGNs attain their multi-NT identity very early in development and maintain it throughout life.

To learn whether the number of CGN vTel^y321^ decreases along the rostro-caudal axis during development, we quantified the proportion of CGN vTel^y321^ neurons at 2 and 4 dpf at rostral, medial, and caudal telencephalon positions. Close to 80% of vTel^y321^ neurons are CGNs at 2 (74.8%, *n* = 4) and 4 dpf (85.9, *n* = 4) across all locations examined (Fig. 3D, Table 2). Though the general trend between 2 and 4 dpf is an increase in the percentage of CGN vTel^y321^ neurons, the increase is not statistically significant (T_(19.8)_ = − 0.59, *p* = 0.55, *n* = 8). As in the adult, there is a general decrease in the percentage of vTel^y321^ neurons that are CGN multi-transmitter neurons in the rostral to caudal direction (Fig. 3D). As with the adult, the proportion of vTel^y321^ neurons that are positive for only GABA or *vachtb* was much smaller. At 2 dpf, 18% of vTel^y321^ neurons were solely GABAergic across all positions, and 21% were solely *vachtb* positive (Table 2). At 4 dpf, 7.2% of GFP positive neurons were solely GABAergic, whereas 13.6% were solely *vachtb* positive (Table 2). Again, these proportions increased from rostral to caudal. We conclude that vTel^*y321*^ neurons are CGNs from very early in development. Overall, the trend we observed in the proportion of vTel^y321^ neurons that are CGNs and non-CGNs (i.e., GABAergic or *vachtb* positive only) in adults is replicated at 2 and 4 dpf, suggesting that NT identity does not switch across the lifetime of these neurons.

### LIMTF genes are expressed in vTel^y321^ neurons throughout development

**Fig. 4:**
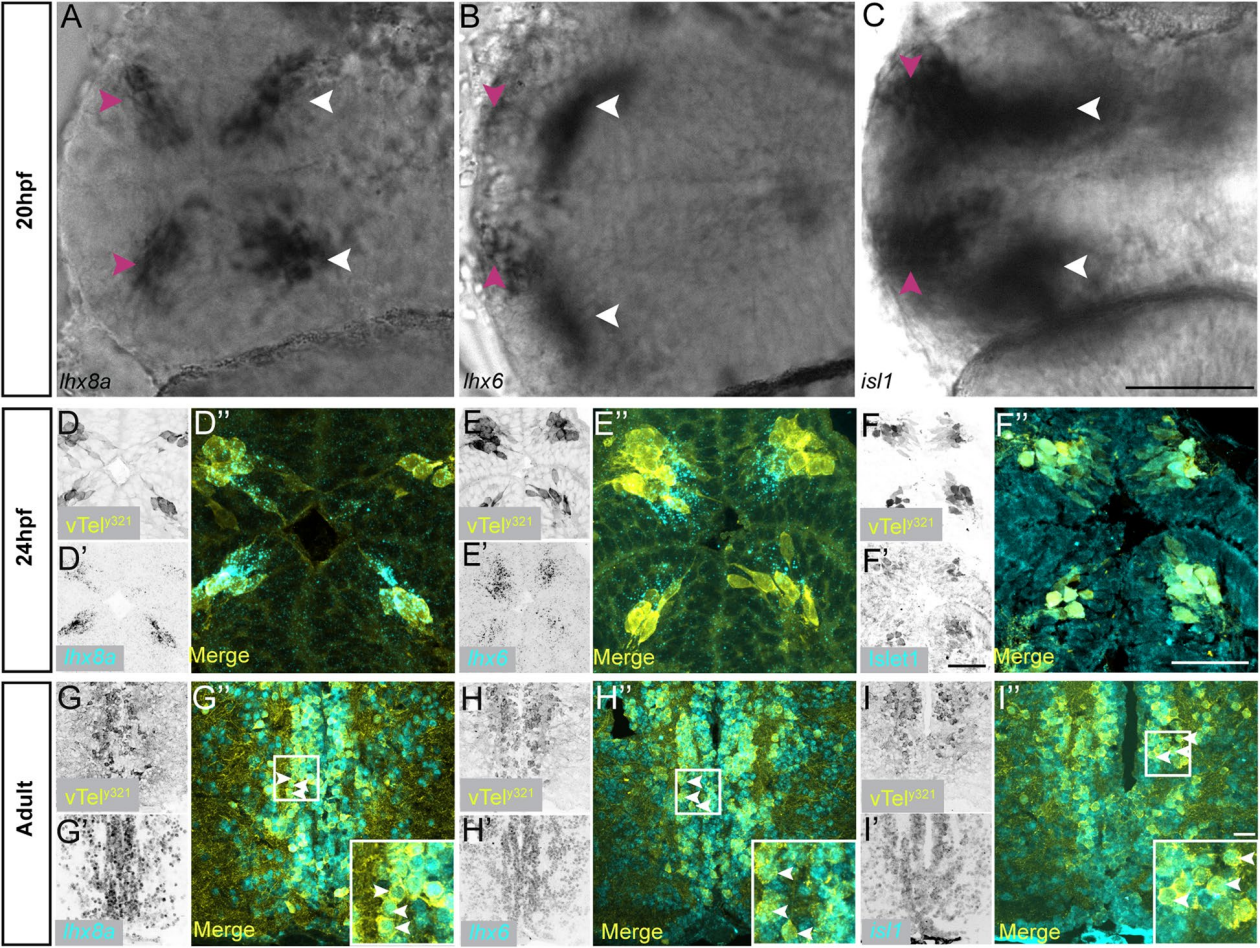
LIMTF genes are expressed throughout development. **A-C** Wholemount chromogenic ISH in 20 hpf embryos showing expression of *lhx8a*, *lhx6* and *isl1* (left to right). Arrows label the principal foci of LIMTF gene expression in the brain. Magenta arrows denote telencephalic clusters and white arrows denote diencephalic clusters respectively at that timepoint. *islet1* is more broadly expressed compared to *lhx8a* and *lhx6*. **D-F** 24 hpf larval forebrain transverse sections showing LIMTF gene expression in vTel^y321^ neurons. **D ***lhx8a* mRNA expression in vTel^y321^ neurons. **E ***lhx6* mRNA expression in vTel^y321^ neurons. **F** islet1 protein expression in vTel^y321^ neurons*. ***G-I**. Coronal sections of the adult ventral forebrain showing (left to right) expression of *lhx8a*, *lhx6* and *isl1* in vTel^y321^ neurons. Selected neurons that express the representative transcripts are denoted by white arrows. (Scale bars: A-C = 50 μm; scale bar in R’ represents D, D′, E, E’, F, F′, G, G’, I, I′ = 30 μm; scale bar in F″ represents D″, E”, F″, G”, H″, I″ = 30 μm)

Two LIMTF genes, *lhx6* and *lhx8a*, are highly expressed in vTel^y321^ neurons (Fig. 1C, D, E). These genes show the highest DE between GFP positive and GFP negative neurons (Additional Table 1). To determine when these transcription factors were first expressed, we crossed *Et*^*y321*^ [*Et (rex2-scp1:gal4ff)y321*] and *Tg (UAS:GFP)* fish and performed ISH and IHC on their offspring to detect forebrain expression of LIMTF genes. The earliest time point at which we detected LIMTFs was approximately 20 hpf (*n* = 12) (Fig. 4A, B); at this stage, *lhx6* and *lhx8a* forebrain expression is restricted to four principal clusters. These clusters have previously been described for *lhx8a* as telencephalic (Vv and Vd) and diencephalic (parvocellular preoptic area, PPa) clusters [24]. It is likely that these clusters give rise to the population of neurons later marked by expression of the transgene vTel^y321^. Indeed, at 24 hpf, expression of *lhx8a* and *lhx6* largely overlapped with GFP expression from the Et^*y321*^ driver (Fig. 4D, D’, 4D”, 4E, 4E’, 4E”). ISH labeling in adult brain sections revealed that expression for these LIMTFs is maintained for the lifetime of the vTel^y321^ neurons (Fig. 4G, G’, 4G”, 4H, 4H’, 4H”).

There is evidence that during development, another LIMTF gene, *isl1*, is expressed in the ventral floor plate region of the telencephalon [25, 26]. Our bulk sequencing data confirmed that *isl1* is significantly DE in vTel^y321^ neurons (Additional Table 1). In addition, since *isl1* expression is typically used as a marker for cholinergic neurons [26] and we know that vTel^y321^ neurons are cholinergic, we asked whether *isl1* expression is detectable in vTel^y321^ neurons, and if so, when. *isl1* expression was detected at 20 hpf in the embryonic forebrain in similar clusters (Fig. 4C) as *lhx6* and *lhx8a.* Labeling at 24 hpf, once GFP expression from the Et^*y321*^ driver is visible, confirms that, similar to *lhx6* and *lhx8* (Fig. 4D-E”), *isl1* is also expressed within vTel^y321^ neurons (Fig. 4D, D’, 4D”, 4E, 4E’, 4E”, 4F, 4F’, 4F”). We also established that this expression is constant throughout development and can be detected in vTel^y321^ neurons in adult telencephalon (Fig. 4I, I’, 4I”). Together, these observations indicate that the 3 LIMTF genes are reliable in identifying vTel^y321^ neurons and that they are expressed throughout the life of those neurons, beginning by the end of the first day of development.

### The vTel^y321^ “transcriptomic fingerprint” is evolutionarily conserved

**Fig. 5:**
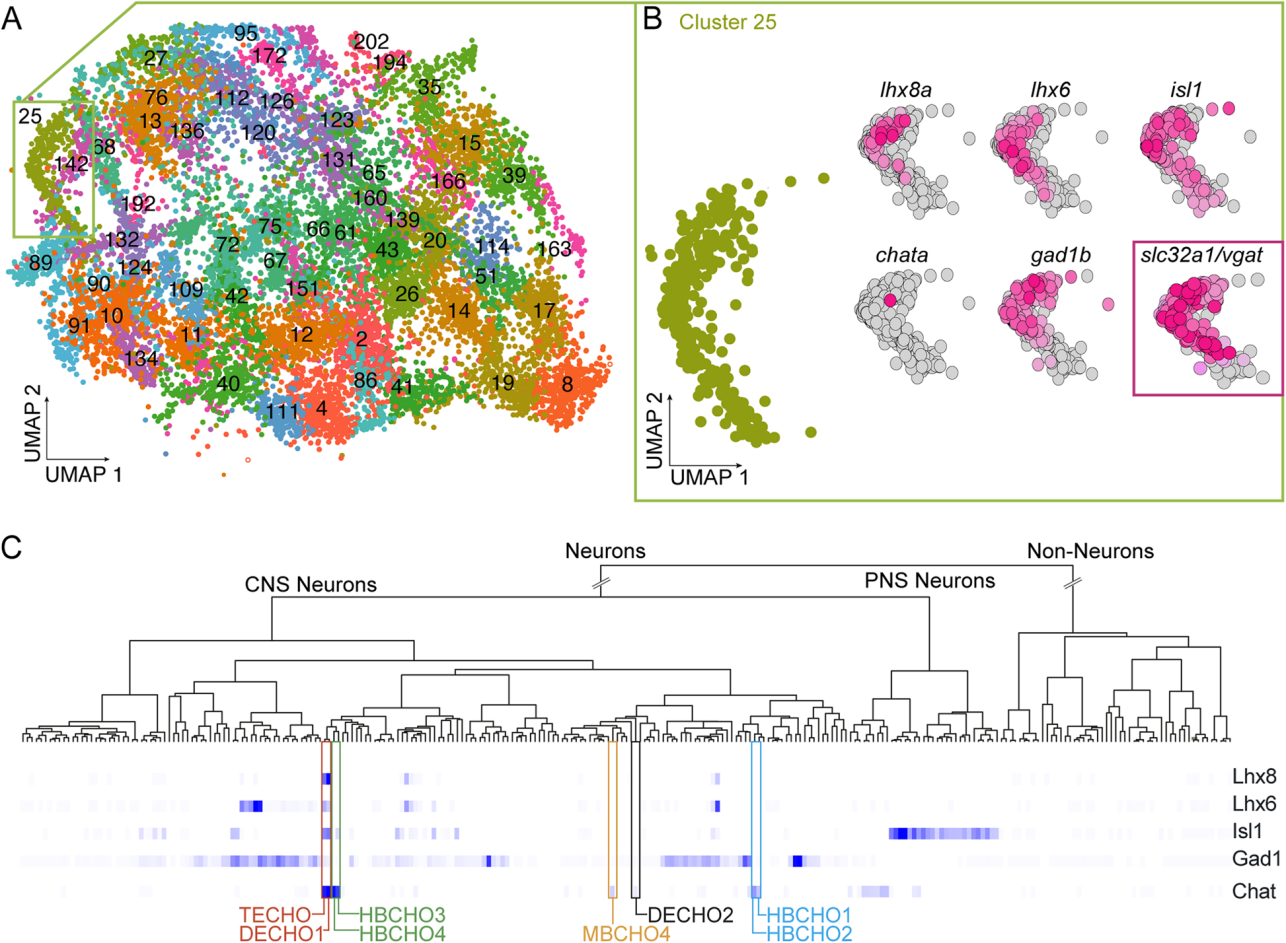
vTel^y321^ “transcriptomic fingerprint” is evolutionarily conserved. **A** Single cell RNA-sequencing data from Atlas of Zebrafish Development plotted using Uniform Manifold Approximation and Projection (UMAP) algorithm. The atlas consists of 220 computationally identified clusters and each of the clusters were annotated using cell-type information from previously identified marker genes from the Zebrafish Information Network (ZIFN). **B** “Transcriptomic fingerprint” expressed genes map onto Cluster 25 of the Atlas and their expression is plotted in UMAP space. *Vgat* expression in Cluster 25 included as a corroboratory marker. **C** Search results from the mousebrain.org online scRNAseq Atlas show that combined expression of *Lhx6*, *Isl1*, *Lhx8, Gad1* and *Chat* maps onto telencephalic (TECHO) and diencephalic (DECHO1) clusters in the mouse brain. Other cholinergic clusters HBCHO1–4, MBCHO4, and DECHO2 do not express the full transcriptomic fingerprint

Multi-NT neurons are a common feature across vertebrates [27]. However, a major challenge is locating anatomically, transcriptionally and functionally homologous neurons in different vertebrate species. Locating homologous neurons across the vertebrate phyla will enable us to better understand the phylogeny of specific behaviors regulated by multi-transmitter neurons. We were thus interested in examining whether vTel^y321^ neurons are conserved in other vertebrates and whether they can be identified by transcriptional homology. To do this, we examined expression of the LIMTF genes, *lhx8a*, *lhx6*, and *isl1*, and the NT pathway genes *gad1* and *chat*, in existing cell sequencing databases*.* First, we compared our bulk RNAseq-data with single cell RNA-sequencing data from 1, 2 and 5 dpf zebrafish, which was compiled into the Single Cell Transcriptome Atlas of Zebrafish Development ([28]; https://cells.ucsc.edu/?ds=zebrafish-dev). The atlas consists of 220 computationally identified clusters and each of the clusters were annotated using cell-type information from previously identified marker genes from the Zebrafish Information Network (ZFIN). Based on expression of the transcriptomic fingerprint from our bulk RNAseq experiments described above, vTel^y321^ neurons map to Cluster 25 of the scRNA-seq Atlas (Fig. 5A). Our dataset of enhanced and statistically significant DE genes was shared with 654 genes from Cluster 25, and therefore served as additional validation of our FACS-based transcriptomic analysis. On further analysis, we established that 212 of the 654 shared genes were classified as forebrain-specific using the ZFIN classification.

We turned our attention to the key genes in our proposed transcriptomic fingerprint, such as the LIMTF genes, and found that all 3 of these genes are significantly expressed in Cluster 25 (Fig. 5B). Interestingly, the ohnolog for *lhx8a*, *lhx8b* was also highly expressed in the shared cluster. The data also reveal high expression of GABAergic NT markers such as the glutamate decarboxylase genes (*gad1b*, *gad2)* and other GABAergic markers such as vesicular GABA transporter (*vgat)*, and the *GABA Transporter 1* (*gat1*). There was low expression of *chata* across Cluster 25, however, we previously detected its ohnolog *chatb* in vTel^y321^ neurons using ISH (Additional Fig. 1). Unfortunately, *chatb* is not annotated in the Atlas gene list due to inconsistencies in genome alignment, therefore we were unable to assess its expression via transcriptome analysis. Nevertheless, we felt confident utilizing *chata* in our analysis because there were also other cholinergic markers that we obtained through bulk RNA-sequencing.

Next, utilizing the three murine LIMTF genes (*Lhx6, Lhx8, Isl1*) and murine NT marker genes (*Gad1* and *Chat)*, we searched for a homologous population of neurons in mouse using the Linnarsson Lab Mouse Brain Atlas (http://mousebrain.org). The Mouse Brain Atlas consists of 265 clusters segregated based on expression of tissue specific transcripts. Using the online atlas, we located two similar clusters of neurons in the telencephalon and diencephalon (Fig. 5C) denoted TECHO and DECHO1. These clusters correlate well with our data in which the LIMTF genes are expressed in two clusters: telencephalic and diencephalic (Fig. 4A-C). To test whether our transcriptomic fingerprint was unique to telencephalic and diencephalic clusters, we searched through the mouse brain atlas for other cholinergic clusters and asked whether they bore the same signature. There were six other clusters that were classified as cholinergic neurons, four of these are in the hindbrain (HBCHO1–4), one located in the midbrain (MBCHO4), and one located in the diencephalon (DECHO2) (Fig. 5C). None of these clusters expressed all the genes in our transcriptomic fingerprint, suggesting that the fingerprint is highly specific for CGNs homologous to vTel^y321^ neurons.

## Discussion

We discovered that a transgenically-defined group of telencephalic neurons required for social orienting behavior in zebrafish co-express ACh and GABA. These neurons, which we refer to as vTel^y321^ neurons, co-express these NTs throughout life from as early as 24 hpf through adulthood. We also discovered that these neurons are marked by the combined expression of three LIMTF encoding genes, *isl1*, *lhx8a* and *lhx6* which are expressed starting prior to development of the multi-NT phenotype through adulthood. Further, by combining expression of the three LIMTFs and specific NT markers, we obtained a minimal transcriptomic fingerprint that can be used to track development of these neurons. In future studies, it will be of interest to determine whether these LIMTFs help define the multi-NT phenotype of this cell type using loss-of-function experiments. In addition, we utilized our transcriptomic fingerprint to identify a homologous population of neurons in the murine forebrain.

Four broad conclusions can be made from our study: (1) A minimal transcriptomic fingerprint can be applied to identify homologous populations across species. (2) Homologous populations of neurons in the basal forebrain expressing ACh and GABA may perform similar functions in teleosts and mammals, (3) the high abundance of multi-NT neurons in vertebrate brains requires a concerted effort to understand how dual NT release functions within specific circuits, and (4) the release of multiple NTs from the same neuronal population confounds pharmacological approaches to multiple neurological conditions. Here we discuss these conclusions.

In this study, we identified a minimal combination of transcription factors that identify a specific cluster of neurons in the mouse brain. A similar approach showed that combinations of homeobox transcription factors are highly specific in defining neuronal sub-groups [29]. Therefore, it will be interesting to determine whether our combined approach can be applied to entire single-cell RNAseq datasets in a high throughput and automated fashion to identify neuronal cell types across species. This will be challenging due to incongruences in gene orthology, particularly for more distantly related species. However, with increasing gene ontology annotation in genome servers, these challenges should soon be remedied [30]. In addition, fingerprints with a minimal number of defining genes might also serve as a basis to design intersectional genetic approaches with which to target transgene expression to defined neuronal populations [31].

The identification of homologous populations of cholinergic and GABAergic neurons in the zebrafish and mouse forebrain raises the question as to whether these neurons also share functional homology. The expression of the three LIMTF encoding genes (*lhx6, lhx8, isl1*), together with *nkx2.1*, another homeodomain transcription factor encoding gene enriched in vTel^y321^ neurons (Fig. 1E and Additional Table 1), marks the medial ganglionic eminence (MGE) in the developing rodent brain. Indeed, *Lhx8* and *Lhx6* co-expression is critical for murine MGE and forebrain neuron development [17, 32, 33]. Zebrafish vTel^y321^ neuron transcriptional profiles map onto the profiles of TECHO and DECHO1 clusters in the mouse forebrain. To our knowledge there have been no functional studies of the TECHO or DECHO1 neuron clusters. However, the DECHO1 diencephalic cluster includes the medial septal nucleus, diagonal band nucleus and nucleus basalis of Meynert (nbM). The nbM provides the largest set of cholinergic inputs to the neocortex [34]. Together with the diagonal band nucleus, the nbM is implicated in evaluating stimuli for their level of significance [34], whereas the septal nuclei are involved in various aspects of social behavior in rodents [7, 35, 36]. Therefore, it is highly possible that vTel^y321^ neurons share common functions with TECHO and DECHO1 neurons in modulating aspects of social behavior. Future studies to interrogate the level to which vTel^y321^ neuron functions are mirrored by the homologous cells in rodents will be extremely informative.

We observed that vesicular transporters for both ACh and GABA are expressed in vTel^y321^ neurons, suggesting that synaptic vesicles in these neurons are capable of being filled with these NTs. A next step to understanding the functions of these neurons within their circuits would be to investigate the possible mechanisms of release: co-transmission with mixed vesicle populations, or co-release of both NTs from the same vesicle. Release of both excitatory and inhibitory NTs at the same time, and potentially at the same synapses, presents an interesting problem: how is a consistent signal transmitted to downstream neurons and what is that signal? In addition to the complexity of releasing more than one neurotransmitter, there is the question of whether two neurotransmitters in the same synapse influence each other’s signaling [37]. In some instances, multi-NT neurons might act as pattern generators with alternate release of excitatory and inhibitory NTs; Lozovaya et al. [12] described a population of CGNs in the mouse striatum for which this appears to be the case. This mechanism is perhaps one of the ways that co-transmission and/or co-release of NTs likely serves an important role in the optimization and maintenance of spatio-temporal patterns of neurotransmission critical for the refinement of behavior [6]. So far, only a function for the release of GABA from the vTel^y321^ neurons has been documented: they inhibit the release of oxytocin in response to social odor cues, thereby reducing defensive behavior [38]. It will be interesting to see how ACh release is incorporated into this circuit, or whether it mediates a completely different functional output.

Co-transmission is found in circuits that are involved in Parkinson [12] and Huntington diseases [4]. However, our understanding of the functional implications of multi-transmission in the pathogenesis and treatment of neurological diseases is still very limited. Current pharmacological approaches for neurological disorders have limited efficacy and though there could be a multitude of factors behind this, it cannot be dismissed that pharmacological approaches fail to mimic the normal endogenous release of (co) transmitters or potential neurotransmitter interactions [4]. Neurotransmitter synergy at specific synapses has been shown to impact aversion, negative-reward and drug dependence [39, 40]. Given that vesicular transporters can load neurotransmitters into single vesicles [6, 37, 41–44], there are potential complications when administering therapeutic agents that target the biosynthesis and release of a single neurotransmitter. Thus, administration of therapeutic agents which do not factor in the multi-transmitter phenotype of specific target neurons could limit efficacy of many neurological drugs.

We conclude that the approach we used to obtain a minimal transcriptomic fingerprint for a set of neurons that modulate social orienting in zebrafish is a reliable method that can be translated to other neuronal populations in other vertebrate species. It enabled us to locate transcriptionally-overlapping neurons in the mouse brain with great specificity. The convergence of gene expression in our fingerprint between zebrafish and mouse could enable comparative functional studies across different vertebrates. In conclusion, the conservation of NT phenotype, gene expression, and functional overlap between vTel^y321^ neurons and murine neuron clusters constitute a valuable platform to investigate the ontogeny of social behavior.

## Methods

### Zebrafish husbandry

All zebrafish embryos, larvae, and adults were raised and maintained at 28.5 °C according to standard protocols [45]. Lines used were AB/Tübingen, *Et*^*y321*^*[Et (rex2-scp1:gal4ff)y321], Tg (mnx1:GAL4)* and *Tg (UAS:GFP)*. All procedures were approved by the University of Oregon Institutional Animal Care and Use Committee.

### Tissue dissection

Forebrains (cut right behind the eyes, see Fig. 1A) were collected from a random sample of 80 7 dpf larvae expressing GFP from the Et^y321^ enhancer trap line and placed into 250-500 μL Neurobasal medium on ice (*n* = 80). Samples were dissociated using the Worthington Papain Dissociation System (Catalog #: LK003150). Briefly, forebrains were spun at 700 g for 5 minutes at RT. Neurobasal medium was removed, Papain + DNase mix was added, and samples were incubated for 30–35 min at RT with constant agitation. Cells were then dissociated further via pipetting until the mixture was homogeneous. Cells were then spun down at 700 g for 5 min at RT. Supernatant was removed, cells were resuspended in stop solution, and incubated with constant agitation for 5 min. Cells were then washed 2x in wash solution (0.22 μM filtered glucose, 1 M hepes, Fetal Bovine Serum, and Dulbecco’s Phosphate Buffered Saline), filtered through a 35 μm strainer (Catalog #:352235), and placed on ice. 1 μL Ghost Red 780 Viability Dye (Tonbo Biosciences, Catalog # 13–0865-T100) was added to 1 mL of wash solution in each tube to resuspend the cells. Cells were sorted within 4 hr. of starting the dissection; longer than this resulted in reduced viability.

### FACS

Fluorescent Activated Cell Sorting (FACS) was performed on a Sony® SH800. Forward scatter (FSC) was set at 16, back scatter (BSC) at 34–36%, and a 120 μm chip used for all sorting. Negative controls (GFP (−) cells) were used to set thresholds to control for green autofluorescence prior to sorting. Positive controls for dead cells (cells treated with ethanol prior to sorting) were used to set thresholds for cell viability dye prior to sorting. Once control thresholds were set, 100,000 cells per sample were sorted directly into 3 mL of Trizol-LS®, ensuring the ratio of cells and sheath fluid to Trizol-LS® was 1:3. Three samples of GFP (−) cells (*n* = 3) and three samples of GFP (+) cells (*n* = 3) were collected for RNAseq.

### RNAseq

RNA collected from sorted cells was first quantified and checked for quality on an Agilent Fragment Analyzer System. Only samples that had a RNA Quality Score (RQN) value of 8 or higher were used for sequencing. RNA libraries were created using the NuGen mRNA Selection Module (Tecan Genomics, Catalog #:0408–32) according to the kit protocol. Libraries were analyzed in Next Generation Sequencing (NGS) mode on an Agilent Fragment Analyzer to ensure good quality cDNA for sequencing. Libraries were then run in single-read sequencing on an Illumina HiSeq 4000®.

### In situ hybridization

Randomly selected adult *Et*^*y321*^ zebrafish (2–12 months, *n* = 4), embryos (16–48 hpf, *n* = 20) and larvae (2–7 dpf, *n* = 28 [4 samples per developmental stage]) were euthanized in ice water, decapitated, and placed in 4% PFA for 1–1.5 hours, before brains were removed and fixed overnight in 4% PFA at RT. After fixation, brains were rinsed 3x in PBS, dehydrated in 20% sucrose in PBS for 24 hours, and cryosectioned after mounting in agarose. RNA in situ hybridization on 16 mm brain sections was carried out according to the protocol of Yan et al. [46], using digoxigenin labeled probes for *lhx8a, gad2*, *gad1b*, *vachtb* and *chatb.* Sections were incubated overnight at 65 °C with 200 ng of probe per section. The following day, sections were washed in a graded series of 5x saline sodium citrate (SSC) with 50% formamide to 2x SSC ending in an incubation step with anti-digoxygenin Fab fragments (Roche) suspended in 1 M Tris HCl pH 7.5, 5 M NaCl and 0.25% Tween 20 (TNT) with Perkin Elmer® block buffer overnight at 4 °C. Sections were washed 8x for 10 min each in TNT and incubated for 5 min in the dark with Perkin Elmer Amplification Diluent, then incubated for 1 hr. at room temperature in the dark with Cy3 for subsequent visualization. Endogenous peroxidase activity was quenched through incubation in 2% hydrogen peroxide in TNT for 1 hr. Sections were first incubated in primary antibodies (chicken anti-GFP, 1:500, Aves Laboratories) and then secondary antibody (goat anti-chicken IgY-488, 1:500, Molecular Probes) overnight in 0.25% PBS Tween. Sections were imaged on a Leica DMI8-CS confocal fluorescence microscope using a 40x objective. See Key Resource Table 1 in Additional File 1 for more information on antibodies and Key Resource Table 2 for accession numbers and references for ISH probes.

### Immunolabeling

Sections were washed 3x for 5 min per wash in PBS followed by a single wash in 0.1% TritonX in PBS (PBST_x_) to permeabilize tissue. The sections were then incubated in block buffer (0.1% PBST_x_ 2% Bovine Serum Albumen and 5% Normal Goat Serum) for a minimum of 2 h and then incubated in appropriate primary antibodies overnight at 4 °C. Primary antibodies were washed off using 0.1% PBST_x_ buffer before incubation with secondary antibodies in block buffer overnight at 4 °C. Excess secondary antibodies were washed off using 0.1% PBST_x_ before applying DAPI Fluoromount-G® (Southern Biotech) and coverslips overnight in the dark at room temperature. To validate IHC, ISH with *gad1b* and *gad2* ISH probes revealed more than 90% overlap with GABA antibody labeling (Additional Fig. 2). Specificity of the antibody was confirmed by testing on spinal cord preparations of intact larvae and sections (Additional Fig. 3). See Key Resource Table 1 in Additional File 1 for more information on antibodies.

### Cell quantification and analysis

Regions of the telencephalon were selected for quantification using the following landmarks (see Fig. 2B):

(i). Rostral telencephalon (RT), at the junction of the ventral telencephalon and olfactory bulb. In this position, vTel^y321^ neurons occupy the ventral and medial portions of the telencephalon including Vv and Vd nuclei.
(ii). Mid-telencephalon (MT), at the anterior commissure (ac). In this position, vTel^y321^ neurons occupy the anterior part of the parvocellular preoptic nucleus and the supracommisural nucleus (Vs) of the ventral telencephalon.
(iii). Caudal telencephalon (CT), at the junction of the forebrain and optic tectum. In this position vTel^y321^ neurons occupy the ventral and medial portions of the forebrain, forming a strip of cells adjacent to the midline that sweeps dorsally and laterally into the dorsal portion of the telencephalon. However, in our observations and quantifications we focused on cells in the ventral telencephalon (subpallium).

Co-expression of GFP, *chatb* (*n* = 4) and *vachtb* (*n* = 4) was quantified using ImagePro Plus version 6.0®. GFP expressing cells were manually identified in a separate image channel and thresholding performed. Data tagged cell templates identified in the GFP channel were loaded onto a second image channel with either *vachtb* or *chatb* labeling*.* In this channel (in this case, *vachtb)*, the fluorescence intensity was assessed for the tagged cell regions. This allowed for quantification of data points which were GFP, *vachtb* or *chatb* positive. Updated points from the combination of GFP and *vachtb* points were then transposed to the third channel (GABA) and the fluorescence intensity for that channel was assessed*.* Each of the channel specific fluorescent intensity data from tagged points was exported into Microsoft Excel. In Excel, the fluorescent intensity for all channels was normalized through subtraction of the sum of the average background intensity (Aμ) and the standard deviation (Aμ + ∂) i.e. [Individual intensity – (Aμ + ∂)]. These data were then used to determine the number of GFP neurons that were GABAergic and/or cholinergic. Plots and graphs for the data were generated in R Studio®. For all developmental stages, a sample size of at least 4 animals was used with quantification of GFP neurons that were cholinergic and GABAergic quantified at the 3 anatomical landmarks (RT, MT, and CT).

### RNAseq data analysis

Raw data files were unzipped and checked for quality using FastQC (Babraham Bioinformatics, version 0.11.8). After ensuring all reads were good quality, cutadapt (v1.18) was used to trim off adapters with the following parameters: -n 3 -O 1 -m 30. Another FastQC was run on these files to ensure the adapter sequences were gone. Trimmomatic (USA Del Lab, version 0.36) was then used to trim off any poor-quality reads in single-end mode. The zebrafish genome index was uploaded (Danio_rerio.GRCz11.dna.primary_assembly.fa.gz) and custom construct (EGFP sequence) were added into the genome index using gmap_build from GMAP-GSNAP package (version 2018-03-25). GSNAP was then used to align the reads from samples to the Zebrafish reference genome. Samtools® (version 1.5) was then used to convert aligned. Fastq reads to .bam files, then sorted and indexed. To generate the counts matrix, htseq-counts (Python version 3.7.0) was used with the following parameters: -f bam --stranded = yes -m intersection-strict. From the counts matrix, protein coding genes were selected using Ensemble Biomart and then used for processing in DESeq2 in R Studio® (R version R.3.5.2). From here, data were analyzed in R using the DESeq2 pipeline instructions provided by Love et al. [47]. After analysis, genes with an adjusted *p*-value of “NA” below 0.05 were removed so that only significant differences were included in the results. For comparisons between bulk RNAseq and single-cell RNAseq datasets, gene lists were analyzed using Excel scripts.

## Supplementary Information

**Additional file 1: Table 1.** LIMTF Gene expression in vTel^y321^ neurons. **Key Resource Table 1. Key Resource Table 2. Additional Fig. 1.***chatb* labelling in vTel^y321^ neurons. Validation of cholinergic identity of vTel^y321^ neurons with *chatb* ISH probes. White arrows point to selected vTel^y321^ neurons expressing both *chatb* and GABA. (Scale bars = 20 μm). **Additional Fig. 2.***gad1b&2* labelling validates GABA immunostaining. g*ad1b* and *gad2* transcripts were labelled in vTel^y321^ neurons and validation staining was performed with GABA antibody. White arrows denote GABA and *gad1b&2* double-labeling, Yellow arrows label vTel^y321^ neurons which are positive for both GABA and *gad1b&2* in the main figure and insert. GABA antibody labelling overlaps extensively with *gad1b&2* confirming its specificity. (Scale bars = 20 μm). **Additional Fig. 3.** GABA antibody labels GABAergic neurons in the larval spinal cord. To test the specificity of the GABA antibody we imaged 24–48 hpf larval spinal cords of the transgenic line *Tg (mnx1:GAL4; UAS:GFP)* which drives GFP expression (yellow) in cholinergic motor neurons. White arrow denotes KA neurons and Cyan arrow points to DoLA neurons which are known GABAergic neurons in the spinal cord. Yellow arrows denote motor neurons (MN). The absence of overlap between the MN and cyan labelling confirms specificity of the GABA antibody. (Scale bars represent 20 μm).

## Data Availability

The data supporting the results of this article are available in the Figshare repository (https://figshare.com/projects/A_Conserved_Transcriptional_Fingerprint_Of_Multi-Neurotransmitter_Neurons_Necessary_For_Social_Behavior/140702) with file names: Vtel_y321_Bulk_RNAseq.xlsx, CGN Data Analysis Summary and 654_Genes_overlap_with_Vtel_y321_FACS.xlsx.

## Abbreviations

ACh: Acetylcholine
CT: Caudal telencephalon
CGN: Cholinergic-GABAergic neuron
DE: Differentially expressed
dpf: Days postfertilization
Et: Enhancer trap
FACS: Fluorescence activated cell sorting
GABA: Gamma-aminobutyric acid
GPCR: G-protein coupled receptor
hpf: Hours postfertilization
IHC: Immunohistochemistry
ISH: In situ hybridization
LIMTF: LIM homeobox transcription factor
MT: Medial telencephalon
NT: Neurotransmitter
RT: Rostral telencephalon
Vd: Dorsal nucleus of the ventral telencephalon (or subpallium)
vTel: Ventral telencephalon
Vv: Ventral nucleus of the ventral telencephalon (or subpallium)
