# Supplementary material for: A conserved transcriptional fingerprint of multi-neurotransmitter neurons necessary for social behavior
Source: BMC Genomics. 2022 Sep 29;23:675. doi: 10.1186/s12864-022-08879-w (PMC9523972; doi:10.1186/s12864-022-08879-w)
Supplement: Supplementary file 1 — Additional file 1: Table 1. LIMTF Gene expression in vTely321 neurons. Key Resource Table 1. Key Resource Table 2. Additional Fig. 1.chatb labelling in vTely321 neurons. Validation of cholinergic identity of vTely321 neurons with chatb ISH probes. White arrows point to selected vTely321 neurons expressing both chatb and GABA. (Scale bars = 20 μm). Additional Fig. 2.gad1b&2 labelling validates GABA immunostaining. gad1b and gad2 transcripts were labelled in vTely321 neurons and validation staining was performed with GABA antibody. White arrows denote GABA and gad1b&2 double-labeling, Yellow arrows label vTely321 neurons which are positive for both GABA and gad1b&2 in the main figure and insert. GABA antibody labelling overlaps extensively with gad1b&2 confirming its specificity. (Scale bars = 20 μm). Additional Fig. 3. GABA antibody labels GABAergic neurons in the larval spinal cord. To test the specificity of the GABA antibody we imaged 24–48 hpf larval spinal cords of the transgenic line Tg (mnx1:GAL4; UAS:GFP) which drives GFP expression (yellow) in cholinergic motor neurons. White arrow denotes KA neurons and Cyan arrow points to DoLA neurons which are known GABAergic neurons in the spinal cord. Yellow arrows denote motor neurons (MN). The absence of overlap between the MN and cyan labelling confirms specificity of the GABA antibody. (Scale bars represent 20 μm). [file 12864_2022_8879_MOESM1_ESM.docx]

**ADDITIONAL TABLES**

**Table 1**

**LIMTF Gene expression in vTel^y321^ neurons**

| Gene | Log2FoldChange | p-value | p-adj |
| --- | --- | --- | --- |
| *lhx8a* | -6.202674283 | 4.83E-72 | 1.00E-67 |
| *lhx6* | -5.126413893 | 1.12E-10 | 1.08E-08 |
| *isl1** | 2.138029493 | 3.61E-17 | 9.86E-15 |
| *nkx2.1* | -3.54286491 | 8.67E-17 | 2.28E-14 |

**isl1* has high differential expression in vTel^y321^ neurons, but is not enriched (positive Log2FoldChange value) since it is widely expressed in other neurons across the brain at this stage of development.

**KEY RESOURCE TABLE 1**

| Target | Manufacturer | Catalog#, clone | Dilution | Species | Immunogen | RRID |
| --- | --- | --- | --- | --- | --- | --- |
| GFP | Aves Lab | GFP-1020 | 1:1000 | Ck | GFP-1020 | AB_10000240 |
| GABA | EMD Millipore | ABN131 | 1:1000 | Rb | GABA mouse, rat IgG | AB_2278931 |
| ChAT | Aves Lab | CAT | 1:500 | Ck | Sequence shared between the mouse (Q03059) and human (P28329) gene products (IgY) | AB_2313537 |
| GFP | Invitrogen | A11120 | 1:1000 | Ms | IgG2a | AB_221568 |
| GFP | Abcam | AB290 | 1:1000 | Rb | IgG | AB_303395 |
| Goat anti-mouse | Invitrogen | A21131 | 1:500 | Gt | IgG2a | AB_2535771 |
| Goat anti-Chicken (FITC) | Aves Lab | F1005 | 1:1000 | Gt | IgY | AB_2313516 |
| Goat anti-Chicken IgY 555 | Invitrogen | A21437 | 1:1000 | Gt | IgY(H+L) | AB_2535858 |
| Goat anti-Rabbit 546 | Invitrogen | A11035 | 1:1000 | Gt | IgG (H+L) | AB_143051 |
| Goat anti-Rabbit 633 | Invitrogen | A21071 | 1:1000 | Gt | IgG(H+L) | AB_2535731 |
| ISLET1/ 2 | Development al Studies Hybridoma  Bank | 39.4D5 | 1:1000 | Rat | Partial protein  Islet-1 & Islet-2 homeobox | AB_2314683 |
|  | | | | | | |

**KEY RESOURCE TABLE 2**

| Probe | Accession number | Source |
| --- | --- | --- |
| *vachtb* | NM_201107.1 | Hong, *et al*.,2013 |
| *chatb* | NM_001291882.1 | Hong, *et al*.,2013 |
| *gad2* | NM_001017708 | Higashijima, S., Mandel, G. and Fetcho, J, (2004) |
| *gad1b* | AB183390 | Higashijima, S, Mandel, G. and Fetcho, J, (2004) |
| *islet1* | NM_130962.1 | Hutchinson, S and Eisen, J, (2006) |
| *lhx8a* | AY664404 | Jackman, *et al.,* (2004). |
| *lhx6* | AY664403 | Jackman, *et al.,* (2004). |
| Microscopy | | |
| Leica TCS SP8 | Confocal Leica  10X/. Leica N/A  40X/1.10 Water Objective Leica 11506357 | https://www.leica-microsystems.com/  products/confocal-microscopes/details/  product/leica-tcs-sp8/ |

**ADDITIONAL FIGURES**


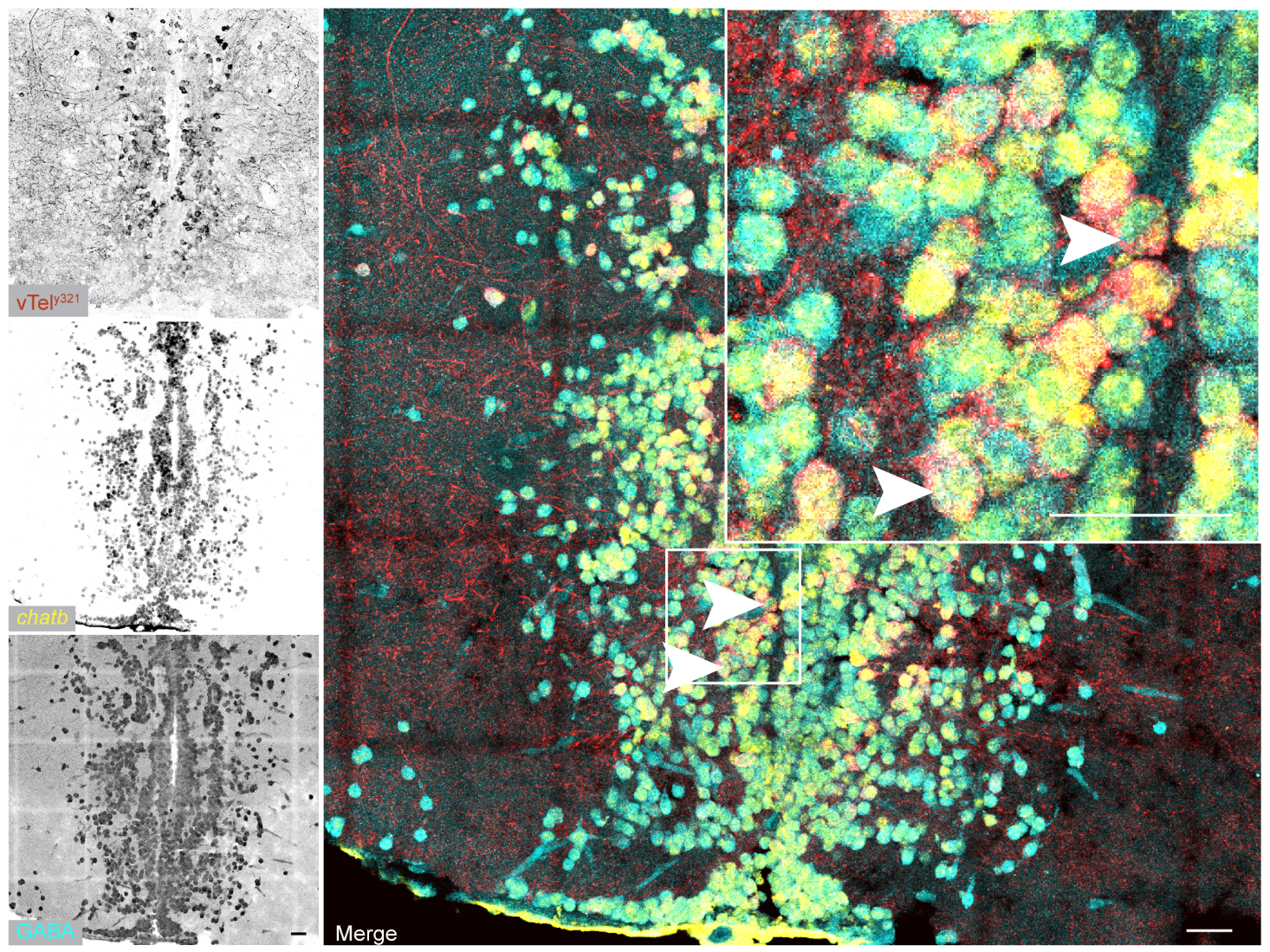


**Additional Fig1: *chatb* labelling in vTel^y321^ neurons.** Validation of cholinergic identity of vTel^y321^ neurons with *chatb* ISH probes. White arrows point to selected vTel^y321^ neurons expressing both *chatb* and GABA. (Scale bars = 20µm).


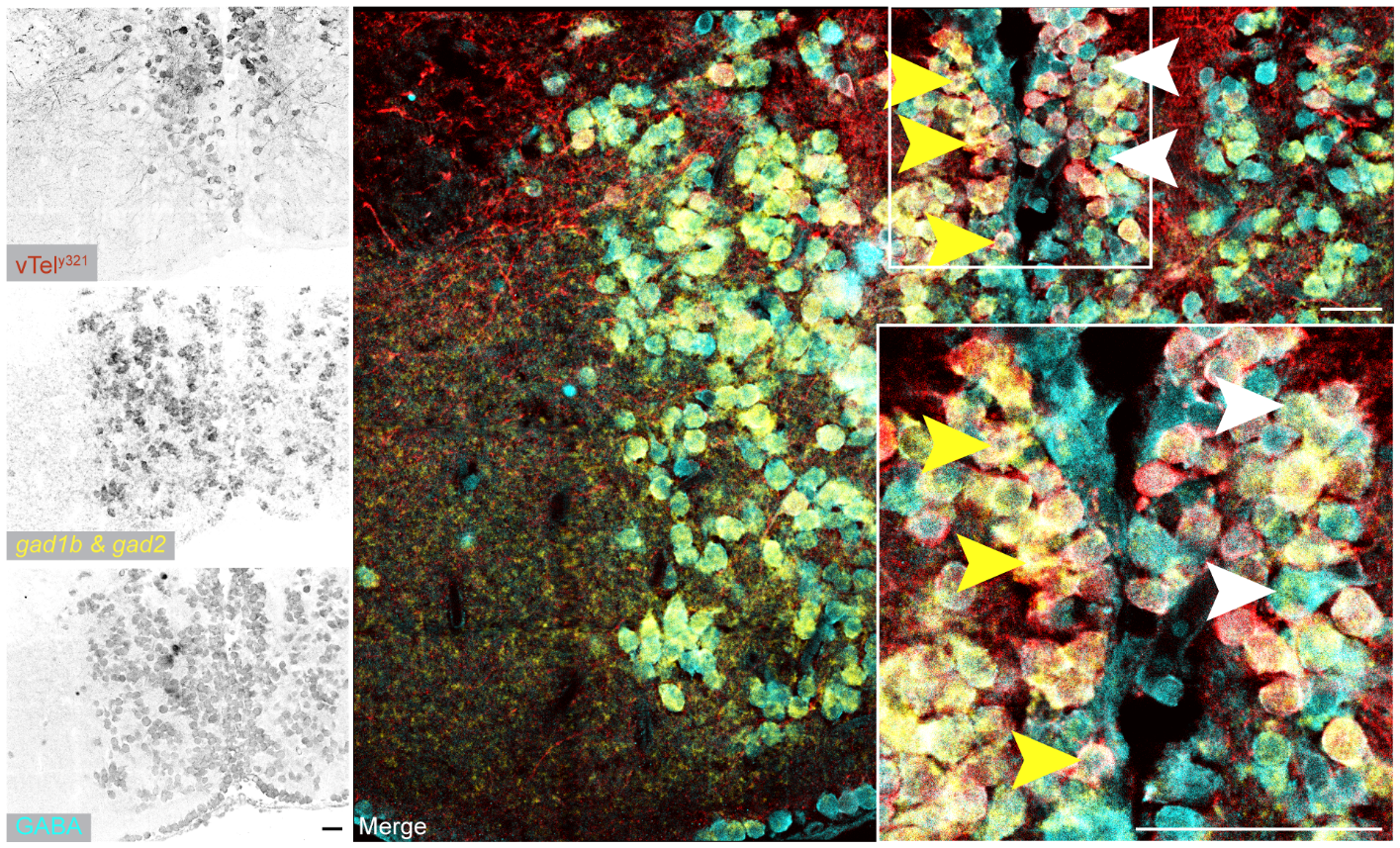


**Additional Fig 2:** ***gad1b&2*** **labelling validates GABA immunostaining**. g*ad1b* and *gad2* transcripts were labelled in vTel^y321^ neurons and validation staining was performed with GABA antibody. White arrows denote GABA and *gad1b&2* double-labeling, Yellow arrows label vTel^y321^ neurons which are positive for both GABA and *gad1b&2* in the main figure and insert. GABA antibody labelling overlaps extensively with *gad1b&2* confirming its specificity. (Scale bars = 20µm).


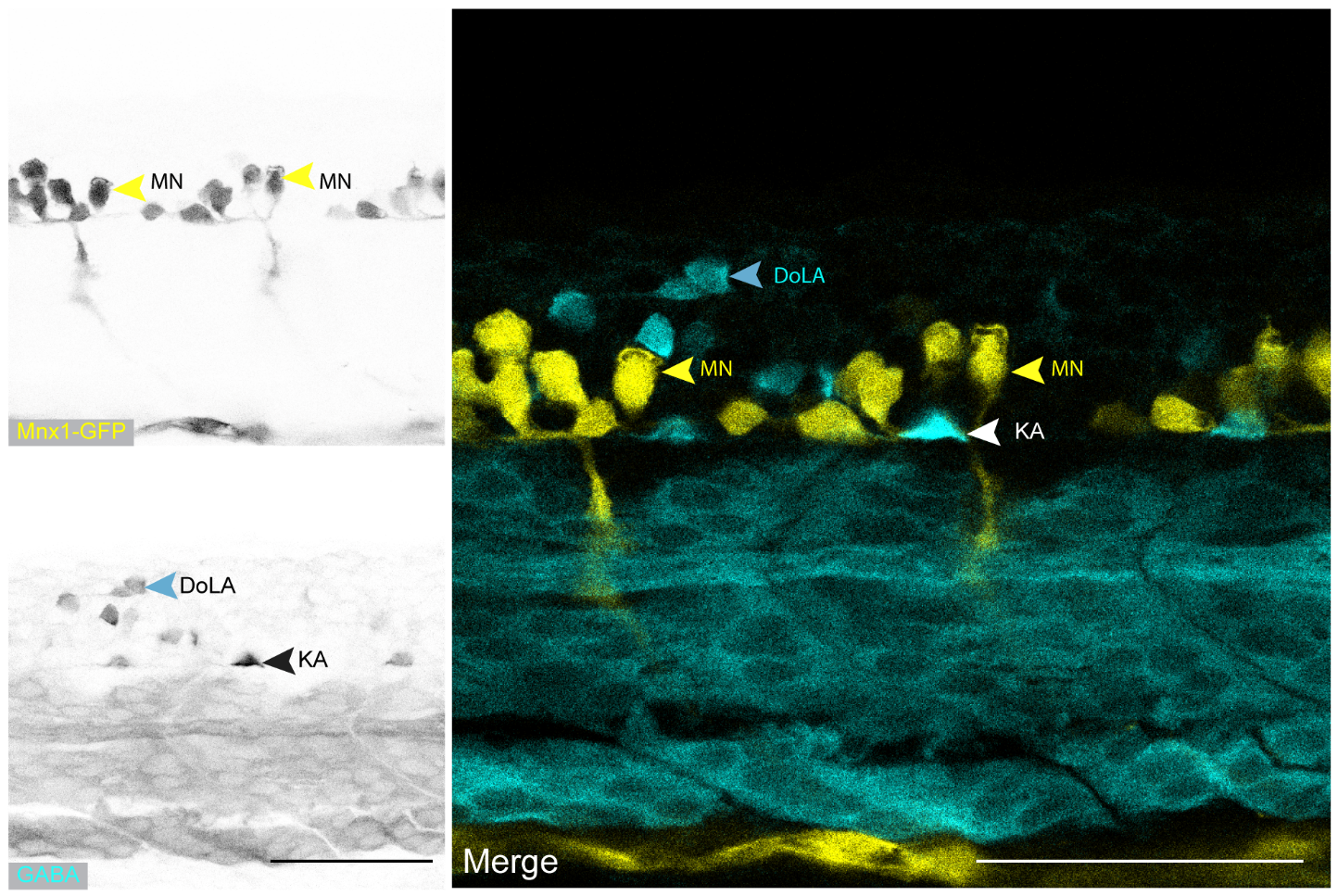


**Additional Fig 3: GABA antibody labels GABAergic neurons in the larval spinal cord.** To test the specificity of the GABA antibody we imaged 24-48 hpf larval spinal cords of the transgenic line *Tg (mnx1:GAL4; UAS:GFP)* which drives GFP expression (yellow) in cholinergic motor neurons. White arrow denotes KA neurons and Cyan arrow points to DoLA neurons which are known GABAergic neurons in the spinal cord. Yellow arrows denote motor neurons (MN). The absence of overlap between the MN and cyan labelling confirms specificity of the GABA antibody. (Scale bars represent 20µm).
